# Synthetic Mucin Gels with Self‐Healing Properties Augment Lubricity and Inhibit HIV‐1 and HSV‐2 Transmission

**DOI:** 10.1002/advs.202203898

**Published:** 2022-09-14

**Authors:** Martin Kretschmer, Rafael Ceña‐Diez, Cosmin Butnarasu, Valentin Silveira, Illia Dobryden, Sonja Visentin, Per Berglund, Anders Sönnerborg, Oliver Lieleg, Thomas Crouzier, Hongji Yan

**Affiliations:** ^1^ School of Engineering and Design, Department of Materials Engineering Technical University of Munich Boltzmannstrasse 15 85748 Garching Germany; ^2^ Center for Protein Assemblies Technical University of Munich Ernst‐Otto‐Fischer Str. 8 85748 Garching Germany; ^3^ Department of Medicine Huddinge Division of Infectious Diseases Karolinska University Hospital Karolinska Institutet, I73 Stockholm 141 86 Sweden; ^4^ Department of Molecular Biotechnology and Health Science University of Turin Turin 10135 Italy; ^5^ Division of Glycoscience Department of Chemistry School of Engineering Sciences in Chemistry Biotechnology and Health KTH Royal Institute of Technology AlbaNova University Center Stockholm 106 91 Sweden; ^6^ Division of Bioeconomy and Health Department of Material and Surface Design RISE Research Institutes of Sweden Malvinas väg 3 Stockholm SE‐114 86 Sweden; ^7^ Department of Industrial Biotechnology School of Engineering Sciences in Chemistry Biotechnology and Health KTH Royal Institute of Technology AlbaNova University Center Stockholm 106 91 Sweden; ^8^ AIMES – Center for the Advancement of Integrated Medical and Engineering Sciences at Karolinska Institutet and KTH Royal Institute of Technology Stockholm Sweden; ^9^ Department of Neuroscience Karolinska Institutet Stockholm SE‐171 77 Sweden

**Keywords:** HIV‐1, HSV‐2, immune suppression, lubricant, mucin hydrogels, self‐healing, strain‐weakening

## Abstract

Mucus is a self‐healing gel that lubricates the moist epithelium and provides protection against viruses by binding to viruses smaller than the gel's mesh size and removing them from the mucosal surface by active mucus turnover. As the primary nonaqueous components of mucus (≈0.2%–5%, wt/v), mucins are critical to this function because the dense arrangement of mucin glycans allows multivalence of binding. Following nature's example, bovine submaxillary mucins (BSMs) are assembled into “mucus‐like” gels (5%, wt/v) by dynamic covalent crosslinking reactions. The gels exhibit transient liquefaction under high shear strain and immediate self‐healing behavior. This study shows that these material properties are essential to provide lubricity. The gels efficiently reduce human immunodeficiency virus type 1 (HIV‐1) and genital herpes virus type 2 (HSV‐2) infectivity for various types of cells. In contrast, simple mucin solutions, which lack the structural makeup, inhibit HIV‐1 significantly less and do not inhibit HSV‐2. Mechanistically, the prophylaxis of HIV‐1 infection by BSM gels is found to be that the gels trap HIV‐1 by binding to the envelope glycoprotein gp120 and suppress cytokine production during viral exposure. Therefore, the authors believe the gels are promising for further development as personal lubricants that can limit viral transmission.

## Graphical abstract

Synthetic mucin gels mimic the material properties of native mucus, exhibiting transient liquefaction under large strain and immediate self‐healing behavior. These gels provide more lubricity and prophylactic activity against HIV compared to simple mucin solution that does not properlumimic native mucus. The gels show promise for further development of personal mucin‐based lubricants that can limit viral transmission.

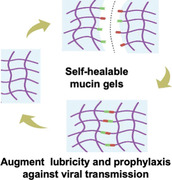

## 1 Introduction

The innate immune system provides crucial protection against viral infections, regardless of the type of virus it encounters and its possible mutations. Mucin glycoproteins are crucial components of innate immunity. These high molar mass, elongated, and highly glycosylated proteins are either located at the cell membrane (i.e., MUC1, MUC4, MUC13, and MUC16) and are involved in the formation of the glycocalyx, or are secreted extracellularly (i.e., MUC2, MUC5AC, MUC5B, and MUC6) to form the mucus gel that covers our moist epithelium. Mucus hydrates, lubricates, and serves as the first line of defense against pathogenic bacteria and viruses, and protects the epithelium from irritation and mechanical stress.^[^ ^1^ ^]^ Alterations or deficiency in mucin expression facilitates virus invasion and subsequent infection.^[^ ^1^ ^]^

Mucus gels can trap viral particles smaller than their mesh size and enable virus clearance through active mucus circulation. Human cervicovaginal mucus (CVM) can effectively trap the human immunodeficiency virus type 1 (HIV‐1) viruses and impede their motion.^[^ ^2^ ^]^ The proposed trapping mechanism is based on binding rather than simple physical size exclusion/steric hindrance, as non‐mucoadhesive nanoparticles (500 nm) can diffuse through the mucus.^[^ ^3^, ^4^ ^]^ Carbohydrate groups on mucins can serve as binding sites for viruses, including *N*‐acetylgalactosamine, fucose, galactose, and sialic acid, which account for 70% of the mass of mucins.^[^ ^5^ ^]^ Among others, sialic acids located at the tip of mucin glycans are binding sites for the various viral proteins^[^ ^6^ ^]^ such as the hemagglutinin of influenza viruses^[^ ^7^ ^]^ and the spike protein of SARS‐CoV‐2.^[^ ^8^ ^]^ Purified porcine gastric mucins have a broad‐spectrum antiviral activity in vitro.^[^ ^9^ ^]^ Purified MUC1 mucins from human breast milk, and salivary MUC5B and MUC7 mucins inhibit HIV‐1.^[^ ^10^, ^11^ ^]^ Mucins inhibit SARS‐CoV‐2 infection in a glycan‐dependent manner.^[^ ^12^ ^]^ Synthetic mucus biomaterials rich in MUC5B mucins can contain influenza A infection more effectively than MUC5AC mucins.^[^ ^13^ ^]^ All in all, mucins and mucin‐derived materials prove to be important contributors to the prophylaxis of viral infections. In addition to mucins, mucus also harbors viral inhibitors that can bind to viruses and slow their spread and infection of epithelial cells.^[^ ^14^, ^15^ ^]^

Mucins have also emerged as vital components of the immune regulatory arsenal.^[^ ^16^ ^]^ Our previous studies have also shown that bovine submaxillary mucin (BSM, MUC5B) gels induce mild activation of the complement system in vitro^[^ ^17^ ^]^ and suppress immune cell recruitment and cytokine production in vivo.^[^ ^10^ ^]^ Furthermore, the immunological activity of BSM gels are influenced by their glycosylation^[^ ^18^ ^]^ and crosslinking architecture.^[^ ^19^ ^]^ BSMs are commercially available mucins that contain 30% by mass of sialic acids, of which ≈70% are Neu5Ac and ≈30% are Neu5Gc.^[^ ^20^, ^21^, ^22^ ^]^ Sialic acids play a vital role in interactions with immune cells^[^ ^23^, ^24^ ^]^ and viruses.^[^ ^12^, ^13^ ^]^ Thus, BSM molecules are one of the most widely used mucins as building blocks for bioactive materials.

According to the Joint United Nations Programme on HIV/AIDS (UNAIDS), 40 million people worldwide are living with HIV‐1, with 2 million new infections per year.^[^ ^25^ ^]^ Despite 30 years of effort, no vaccine against HIV‐1 has been developed. In addition, individuals carrying genital herpes simplex type 2 (HSV‐2) have a three‐ to fourfold increased risk of acquiring HIV‐1.^[^ ^26^, ^27^ ^]^ Therefore, prevention of HSV‐2 infection would indirectly reduce the risk of new HIV infections in individuals. The development of a new prophylactic strategy for both types of viruses is, therefore, a high priority.

The first step in virus entry into target cells occurs through the interaction of viral envelope glycoproteins with specific cell surface receptors. The glycoprotein 120 (gp120) of HIV‐1 interacts with CD4 receptors on mononuclear phagocytic cells and helper T lymphocytes^[^ ^28^, ^29^ ^]^ and subsequently with the coreceptors CCR5 or CXCR4.^[^ ^30^, ^31^ ^]^ The binding of gp120 results in conformational changes in the structure of the viral envelope proteins that allow exposure of the interaction domain of gp120 to the chemokine receptors CCR5 or CXCR4.^[^ ^32^ ^]^ HSV‐2 interacts with cell surface glycosaminoglycans such as unmodified heparin sulfate (HS)^[^ ^33^ ^]^ on mucosal epithelial cells and epidermal keratinocytes.

Local immune activation has a critical impact on HIV‐1 replication.^[^ ^34^ ^]^ Production of proinflammatory cytokines such as TNF*α* increases the risk of HIV‐1 acquisition.^[^ ^35^, ^36^ ^]^ In addition, in clinical studies, upregulation of proinflammatory cytokines in cervicovaginal tissue results in a threefold increase in HIV‐1 acquisition and local replication.^[^ ^36^ ^]^ Therefore, inhibition of cytokine production may help limit HIV‐1 transmission.

In this study, we developed BSM gels that exhibited transient liquefaction under large strain and immediate self‐healing behavior like native mucus gel.^[^ ^37^ ^]^ We investigated whether BSM gels with these properties had “mucus‐like” functions in lubrication and prophylaxis against two types of sexually transmitted viruses: HIV‐1 and HSV‐2, using different types of cells, including primary immune cells. Subsequently, the prophylactic activity against HIV‐1 was demonstrated to be due to binding to HIV‐1 envelope protein gp120 and by dampening the immune cell activation during HIV‐1 exposure. Finally, we propose that synthetic mucin gels with such properties are very promising as lubricants in the biomedical field and as prophylaxis against viral infections in personal lubricants.

## 2 Results and Discussion

### 2.1 Synthesis of BSM Derivatives That Form Dynamic Covalent Carbohydrazide (CDH)‐Hydrazone Bonds

We successfully introduced aldehydes or hydrazides to BSM molecules, respectively. The resulting BSM aldehydes and hydrazides can form dynamic covalent hydrazone bonds when mixed in solution, leading to the formation of BSM gels. We chose COMU coupling chemistry with DIPEA for the synthesis of BSM derivatives because it is safer and more effective than benzotriazole‐based uronium reagents.^[^ ^38^ ^]^ Nucleophilic substitution between the amines and the activated acids occurred in the presence of DIPEA because the hindered base acted as a proton scavenger without competing with the amine as a nucleophile. To synthesize BSM aldehyde derivatives, we first introduced 3‐amino‐1,2‐propanediol to the activated carboxyl groups of BSM to obtain BSM *cis*‐diol derivatives, followed by periodate oxidation^[^ ^38^, ^39^ ^]^ (Figures S1 and S2, Supporting Information). Flexible vicinal diols on the 3‐amino‐1,2‐propanediol residues facilitate selective and efficient oxidation compared to other vicinal diols with *trans* geometry on glycosaminoglycans, thus preserving the native structure.^[^ ^39^ ^]^ The preferential oxidation of the added glycol residues was suggested by the measured^[^ ^40^ ^]^ initial accelerated oxidation kinetics of BSM *cis*‐diol derivatives compared to native BSM (Figure S3, Supporting Information). BSM‐hydrazide derivatives were synthesized by introducing carbohydrazide (CDH)‐hydrazide to activated carboxyl groups of BSM^[^ ^38^ ^]^ (Figure S4, Supporting Information).

Lyophilized BSM derivatives were further analyzed by attenuated total reflectance‐Fourier transform infrared spectroscopy (ATR‐FTIR) and compared with unmodified BSM. The ATR‐FTIR spectrum of BSM‐*cis*‐diol derivatives shows a shift in the peak of amide I at 1647 cm^−1^ (unmodified BSM) to 1633 cm^−1^ and a slight shift in the peak of amide II at 1534 cm^−1^ (unmodified BSM) to 1544 cm^−1^. The peaks of amides I and II also overlap with the O‐H bending peaks of H_2_O, which need to be interpreted with caution. The full width half maximum (FWHM) of the O—H stretching peak at 3284 cm^−1^ increased from 184 to 251 cm^−1^ for the BSM *cis*‐diol derivatives compared with unmodified BSM (Figure S5A, Supporting Information). The ATR‐FTIR spectrum of BSM‐aldehyde derivatives is consistent with that of BSM *cis*‐diol derivatives, as the amide I peak at 1647 cm^−1^ (unmodified BSM) and the amide II peak at 1534 cm^−1^ (unmodified BSM) are shifted to 1637 and 1542 cm^−1^, respectively, compared with BSM *cis*‐diol derivatives (Figure S5B, Supporting Information). However, we could not clearly identify the C=O stretching peak at 1730 cm^−1^ attributed to aldehydes, for the BSM‐aldehyde derivatives because the peak is also present for BSMs. We confirmed the synthesis of BSM‐aldehyde derivatives using Schiff's reagent with periodic acid tests^[^ ^41^ ^]^ (Figure S6, Supporting Information). The BSM aldehyde derivatives contain 9.34 ± 2.01 µmol aldehydes per mg.

The ATR‐FTIR spectrum of BSM‐hydrazide derivatives showed doublet peaks at 3226 and 3317 cm^−1^ (possible peak for N—H stretching) but not for the unmodified BSM (Figure S5C, Supporting Information). Also, the observation of a small kink at 1639 cm^−1^ could be an additional indication of N—H bend or C=N stretching, while there are also two small new peaks at 1199 and 1208 cm^−1^.

**Figure 1:**
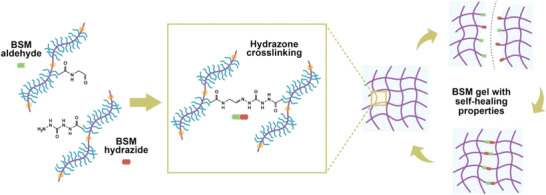
Schematic illustration of the dynamic covalent hydrazone bonds forming BSM gels.

BSM gels (5% wt/v) were obtained by carbodihydrazide (CDH)‐hydrazone crosslinking chemistry (**Figure** **1** and Figure S7, Supporting Information). BSM gels showed two new peaks at 798 and 1260 cm^−1^ compared to BSM aldehyde and BSM hydrazide derivatives. In addition, two peaks specific for hydrazide at 3226 and 3317 cm^−1^ (doublet peak for N—H stretching) disappeared for BSM gels. The data suggest consumption of hydrazide and formation of hydrazone bonds (Figure S5D, Supporting Information).The bonds can break once a high enough stress is applied and regenerate after the stress is removed. This chemistry has been used to form hydrogels with strain‐weakening and self‐healing properties.^[^ ^42^, ^43^, ^44^ ^]^

### 2.2 BSM Gels Formed by Hydrazone Bonds Exhibit Transient Fluidization and Very Good Self‐Healing Behavior

**Figure 2:**
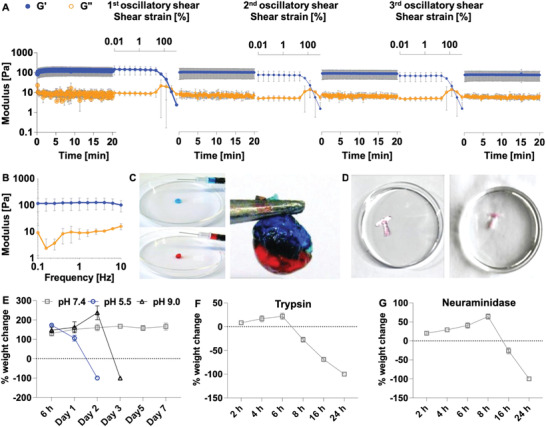
Rheological, strain‐weakening, and self‐healing properties of BSM gels. A) Time‐dependent sweep analyses (linear material response) and strained‐controlled time‐dependent sweep analyses with an increase in oscillatory shear strain from 0.1% to 1000% and a constant oscillation frequency of 1 Hz (nonlinear material response) were performed for three cycles. Each test of the time‐dependent sweep was repeated 5 s after the BSM gels were ruptured by high oscillatory shear strain. B) Frequency‐dependent sweep analyses (linear material response) were performed. C,D) The injectability and self‐healing properties of BSM gels were tested. E–G) The hydrolytic and enzymatic stability of the BSM gels by swelling/disintegration studies. Error bars indicate the standard deviation obtained from *n* = 3 independent measurements.

The viscoelastic properties of BSM gels (5% wt/v) (including gel behavior in the linear response regime as well as liquefaction and self‐healing properties of the gels after exposure to large strains) were studied using a commercial shear rheometer. Time‐dependent sweep analyses showed that stable BSM gels were formed: within a few seconds upon mixing, the material response was dominated by the elastic modulus (*G*′), which reached a plateau‐like state after ≈60 s (**Figure** **2**A). Subsequently, frequency sweeps confirmed that the gels were efficiently and covalently crosslinked: within the range of probed frequencies, no distinct frequency‐dependent viscoelasticity was observed (Figure 2B). BSM gels had an estimated average mesh size (*ξ*) of 38.83 ± 6.67 nm, as calculated based on rubber elastic theory.^[^ ^45^ ^]^

Furthermore, we demonstrated the dynamic nature of the covalent hydrazone bonds‐formed BSM gels by observing a pronounced strain‐weakening behavior (that even results in fluidization of the gel) in combination with a highly efficient and rapid self‐healing behavior. A series of strain‐controlled and time‐dependent sweep analyses transiently reduced the elastic properties of the BSM gels by two orders of magnitude and even led to a state where the material response was dominated by viscous properties (Figure 2A). However, immediately after this fluidization step (in which the material response was transformed from a viscoelastic solid/gel to a viscoelastic liquid), the system autonomously and rapidly recovered close to its original properties within 5 s (Figure 2A). Importantly, this self‐healing behavior not only qualitatively reinstated the gel state of the material but also allowed the system to recover 85% ± 13% of its initial stiffness (Figure 2A). Recovery of elastic behavior occurred even after repeatedly enforcing fluidization, albeit with slightly reduced efficiency (to 76% ± 16% and 74% ± 17% of the initial gel stiffness after the second and third fluidization, respectively). In other words, the BSM gels can reversibly undergo mechanically induced gel‐sol transitions and return to their gel state once the shear load is removed.

We showed that gels can be pushed through a needle (G20) and immediately form stable gels again, so that two stained gels (blue and red) can be quickly fused to form a cohesive gel (Figure 2C). We also showed that BSM gels injected through a needle (G20) can form an intact T‐shape and maintain their integrity in a PBS buffer (Figure 2D). This behavior is very interesting for many applications of this material, as it allows the gel to be easily squeezed out of a bottle, while quickly resuming its gel state at the point of use.^[^ ^42^, ^46^ ^]^ Self‐healing gels can be synthesized by dynamic covalent or non‐covalent bonds.^[^ ^47^, ^48^ ^]^ The choice of dynamic covalent bonds ensures more stable gels with a slower dynamic equilibrium.^[^ ^49^ ^]^ However, the dynamic covalent bonds of CDH‐hydrazone forming BSM gels showed rapid dynamic equilibrium, as evidenced by the rapid self‐healing behavior of the gel.

Next, we investigated the hydrolytic and enzymatic stability of the BSM gels by swelling/disintegration studies, following the previously reported methods.^[^ ^50^ ^]^ The BSM gels initially swelled and then were stable under neutral pH conditions until Day 7 (Figure 2E). The swelling of BSM gels was greater at acidic and basic pH. BSM gels were hydrolyzed on Day 2 and Day 3 under acidic and basic pH conditions, respectively (Figure 2E). BSM gels were degraded more rapidly when immersed in a PBS buffer at pH 7.4 with trypsin (50 µL of 0.5 µg mL^−1^, Figure 2F) compared with 2% neuraminidase (5 × 10^4^ U mL^−1^, Figure 2G) and dissolved after 1 d. For use as personal lubricants, the stable gels are suitable if they are stable for ≈1 d, after which they are excreted. Interestingly, a previous report showed that hyaluronic acid gels prepared with CDH‐hydrazone were much more stable when the gels were incubated in buffers with pH 5.5, 7.4, and 9.0, and in solutions containing enzymes, compared with oxalyldihy (ODH)‐hydrazone and adipoyldihydrazide (ADH)‐hydrazone chemistry.^[^ ^50^ ^]^ CDH‐hydrazone‐BSM gels were significantly less stable under different pH solutions than CDH‐hydrazone‐hyaluronic acid gels.^[^ ^50^ ^]^

### 2.3 BSM Gels That Undergo Fluidization under Large Strain and Rapid Self‐Healing Improve Lubricity

**Figure 3:**
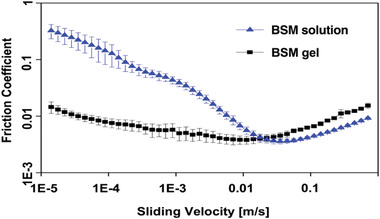
Tribological properties of BSM gels were studied using a commercial shear rheometer equipped with a tribology unit (T‐PTD 200, Anton Paar) with a steel ball‐on‐PDMS cylinder, as reported previously.^[^ ^57^ ^]^ The better lubricity was indicated by the lower friction coefficient of BSM gels (5% wt/v) compared to BSM solutions (5% wt/v). All tribological experiments were conducted at 21 °C and using a solvent trap. A paper towel soaked with water was used in a closed chamber to prevent evaporation of the sample during the experiments. Error bars indicate the standard deviation determined from *n* = 3 independent measurements.

Mucus is a shear‐thinning and self‐healing gel that lubricates our epithelium.^[^ ^37^, ^51^ ^]^ BSM solutions were also shown to lubricate hydrophobic surfaces, such as PDMS. The effect was dependent on intact protein backbone, as suggested by the loss in lubricity of protease‐treated BSMs.^[^ ^52^ ^]^ We therefore wondered whether BSM gels with such properties can replicate this function. We used a commercial shear rheometer equipped with a tribology unit (T‐PTD 200, Anton Paar) and a steel ball‐on‐PDMS cylinder material pairing to compare the lubricity of BSM gels (5% wt/v) and BSM solutions (5% wt/v, Figure S8, Supporting Information). With this setup, we can probe all three possible regimes of lubrication, i.e., boundary lubrication, mixed lubrication, and hydrodynamic lubrication. Interestingly, we obtained a significantly lower friction coefficient in both the boundary lubrication and mixed lubrication regimes when BSM gels were loaded into the tribology unit, compared to BSM solutions used as lubricants (**Figure** **3**). In humans and animals, the first two regimes (boundary and mixed lubrication) are of primary importance, as very high sliding velocities occur only during selected processes such as eye blinking.^[^ ^53^ ^]^ Previous work has shown that solutions of PGM purified in the laboratory lubricate PDMS surfaces better than commercially available mucins and a pure buffer that increases the coefficient of friction by several orders of magnitude.^[^ ^54^, ^55^ ^]^ In the present study, the BSM gels augmented lubricity compared to simple BSM solutions, hence suggesting that gel properties played a vital role in lubrication properties. It could be attributed to the reversible crosslinking between BSM molecules in the BSM gel network that increased the viscosity when gels were liquifacted. The result was consistent with other study showing that polymer‐based gels can lubricat better than their solutions.^[^ ^56^ ^]^ Considering future applications, these results highlight that BSM gels could be very promising as lubricants in the biomedical field.

### 2.4 BSM Gels Increase Prophylactic Efficacy against HIV‐1 and HSV‐2 Infections Compared to BSM Solutions

**Figure 4:**
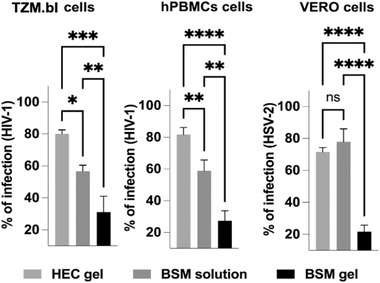
Prophylactic efficacy against HIV‐1 and HSV‐2 infections of BSM gels (5% wt/v), BSM solutions (5% wt/v) and HEC gels (2% wt/v) compared to the control (no protection). The gels or solutions were added to the cells before they were exposed to the viruses and removed after 1 h of virus exposure. BSM gels were the most effective prophylaxis, inhibiting infection of TZM.bl and PHA‐activated hPBMC cells by HIV‐1 (R5‐HIV‐1NL (AD8) isolate) as well as VERO cells by HSV‐2. BSM solutions inhibited only HIV‐1 but not HSV‐2 infection. HIV‐1 infection of hPBMCs was measured indirectly by transferring the supernatant containing newly produced HIV‐1 from hPBMCs after 48 h to TZM.b1 cells. HIV‐1 infection of TZM.b1 cells was measured by luciferase assay. HSV‐2 infection of VERO cells (0.1 MOI, multiplicity of infection) was determined by quantifying the number of viral plaques at 48 h post‐infection. The error bars denote the standard deviation as obtained from *n* = 3 independent experiments. Differences were determined using ordinary one‐way ANOVA tests.

Mucus protects the epithelium from viral infections. Therefore, we wondered whether synthetic BSM gel (5% wt/v) could mimic this prophylactic function against viral infections. The prophylactic efficacy of BSM gels (5% wt/v) was compared with BSM solutions (5% wt/v) and hydroxyethyl‐cellulose (HEC) gels (2% wt/v) in vitro. HEC gels are prepared from Natrosol 250HX Pharm HEC. 2% (wt/v) HEC gels (1.3 MDa) were selected because the elastic modulus is similar to synthetic BSM gels (≈100 Pa) and they have shear‐thinning properties,^[^ ^58^ ^]^ as indicated in the company's instructions for use. To make the gel, HEC polymers were dissolved in a pH 7.4 PBS buffer, and the dissolved HEC polymer particles formed a 3D gel network. The viscoelastic HEC gels have been widely used before as a “universal” placebo in clinical evaluations of vaginal microbicides,^[^ ^59^ ^]^ as well as vehicles for antiviral drugs in microbicides against different viruses, including HIV‐1.^[^ ^60^, ^61^ ^]^ Many microbicides for topical vaginal or rectal prophylaxis against HIV‐1 are in clinical trials, as HIV‐1/AIDS remains the most severe global epidemic.^[^ ^62^, ^63^, ^64^ ^]^ In the present study, the BSM gels increased the prophylactic efficacy against a prototype HIV‐1 CCR5 strain infection of TZM.bl cells compared to BSM solutions only (**Figure** **4**). This prophylactic efficacy of BSM gels was also significantly higher in human peripheral blood‐derived mononuclear cells (hPBMCs) isolated from healthy donors than that of BSM solutions (Figure 4 and Figure S9, Supporting Information). The percentage of infected cells decreased to ≈30% and ≈60%, respectively, when cells were protected with BSM gels (5% wt/v) compared to BSM solutions (5% wt/v).

We also showed that BSM gels could limit HSV‐2 infection of epithelial VERO cells by 80%. In contrast, no protection against HSV‐2 was found with BSM solutions (5% wt/v) (Figure 4). For HEC gels, very limited prophylaxis against HIV‐1 and HSV‐2 was found (Figure 4), likely because HEC molecules have no binding sites for viral protein. The limited prophylaxis against bigger viral particles HSV‐2 could be due to the steric hindrance. HSV‐2 infection causes lifelong infection and increases the risk of acquiring HIV‐1 by three‐ to fourfold.^[^ ^65^ ^]^ As a result, prophylaxis products against both viral types would be more effective in limiting HIV‐1 transmission. We therefore suggest that BSM gels, which temporarily liquefy under high stress and rapidly self‐heal after the stress is removed, could be developed into personal lubricants with a viral prophylactic function. Cervicovaginal mucus (CVM) has been shown to effectively scavenge HIV‐1 at acidic pH but not at neutral or basic pH.^[^ ^4^ ^]^ At ejaculation, the pH of CVM is rapidly neutralized by semen, which has a pH of 7.7 and a substantial pH buffering capacity.^[^ ^66^, ^67^ ^]^ Therefore, the protective role of CVM against HIV‐1 after coitus might be low in real life. Similar conclusions can be drawn for rectal fluids, which also have a neutral pH.^[^ ^68^ ^]^ In this work, we have performed all assays at a close to neutral pH, mimicking the neutral pH state of CVM after coitus.

### 2.5 BSM Binds to HIV‐1 Envelope Glycoprotein 120

**Figure 5:**
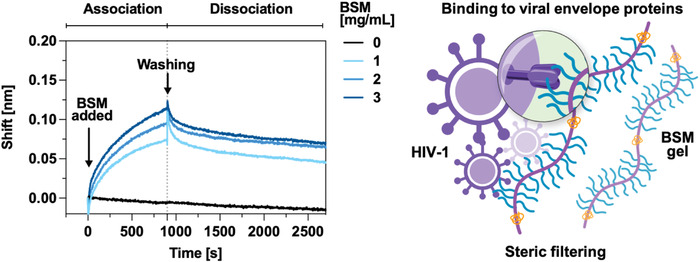
BSM binds to HIV‐1 gp120. Association and dissociation curves showing real‐time binding of BSM to gp120 were obtained using biolayer interferometry (BLI). Concentration‐dependent (0, 1, 2, 3 mg mL^−1^) responsive shifts were observed. The black dotted line indicates the separation of the association and dissociation phases. The results are an average of three independent measurements.

Virus entry occurs through the interaction of viral envelope glycoproteins with specific cell surface receptors. The HIV‐1 gp120 binds to several cell surface receptors, facilitating the internalization of viruses by cells and subsequent infection.^[^ ^28^, ^29^ ^]^ We hypothesized that the increased prophylactic efficacy of BSM gels (5% wt/v) against HIV‐1 infection compared to BSM solutions (5% wt/v) was due to BSM gels mimicking the mucus's viscoelastic properties, thus better trapping viruses if mucins bind to HIV‐1 envelope protein. The mesh size of BSM gels (*ξ*) of 38.83 ± 6.67 nm is much smaller than the diameter of the viral particles (HIV‐1 ≈ 100 nm; HSV‐2 = 160 nm). We propose that BSM gels may trap viruses through a combination of steric filtering and binding to viral envelope proteins. In the present study, we investigated the binding of BSM to gp120 by biolayer interferometry (BLI). The shift (nm) showed an initial fast binding phase and later a slower phase that never reached equilibrium in the association curve, as well as a fast dissociation decay (swoop shape) followed by a slower dissociation decay in the dissociation curve that did not reach the baseline signal as in the association curve, indicating heterogeneous binding profiles between BSM and gp120 (**Figure** **5**). Better fitting of the real‐time binding curve was obtained with a “2:1 heterogeneous ligand binding model” compared to a “1:1 ligand binding model” (Figure S10, Supporting Information). The “2:1 heterogeneous ligand binding model” measures two independent binding interactions, according to the Octet Data Analysis Software instructions. This result suggests that gp120 interacts with BSM via two distinct binding mechanisms and affinities.

The binding area (%), association rate constants (*K*_a_), dissociation rate constants (*K*_d_), and equilibrium dissociation constants (*K*_D_) were calculated using the 2:1 heterogeneous ligand binding (**Table** **1**). The result showed that 76% of the binding surface area of the gp120‐BSM complex was due to primary binding interactions with a lower *K*_a_ (the number of complex molecules formed per second in a unimolar mixture of two components and the “dissociation” rate constant) and *K*_d_ (the fraction of the complex that dissociates per second) than the secondary binding interactions. Two independent binding interactions had similar *K*_D_ values (the ratio of dissociation to association rate constants or the concentration of BSMs required to occupy 50% of the surface ligand sites at equilibrium) of 0.88 ± 0.14 and 1.1 ± 0.37 × 10^−6^ m, respectively.

**Table 1:** Equilibrium dissociation constants (*K*_D_), association rate (*K*_a_), and dissociation rate (*K*_d_) constants, and binding area of BSM to HIV‐1 gp120 were calculated by the Octet Data Analysis software using the “2:1 heterogeneous ligand binding model.” Error bars indicate the standard deviation obtained from *n* = 3 independent experiments

	Primary binding interactions	Secondary binding interactions
Binding surface area [%]	76 (± 4)	24 (± 4)
Rate of association *K*_a_ [m ^−1^ s^−1^]	1.0 (± 0.22) × 10^3^	1.77 (± 1.0) × 10^4^
Rate of dissociation *K*_d_ [s^−1^]	8.7 (± 0.93) × 10^−4^	1.72 (± 3.12) × 10^−2^
Equilibrium dissociation constant *K*_D_ (µm)	0.88 (± 0.14)	1.1 (± 0.37)

### 2.6 BSM Gels Dampen Cytokine Production from hPBMCs during HIV‐1 Exposure

**Figure 6:**
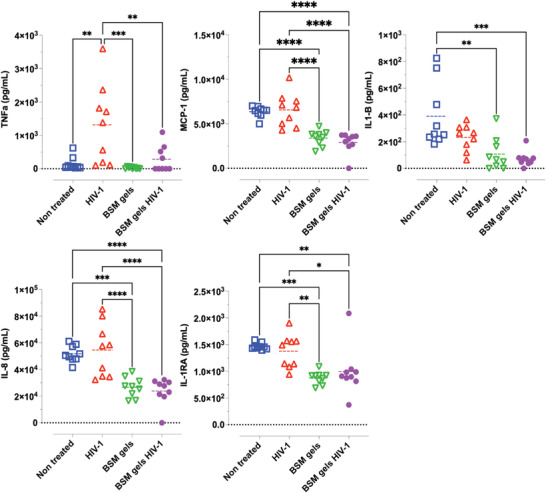
Cytokine secretion from hPBMCs after HIV‐1 exposure with and without protection by BSM gels. Cells were protected with or without BSM gels and then exposed to HIV‐1 viruses for 1 h. Cytokine secretion after 24 h of incubation was quantified using U‐PLEX Inflammatory Panel 1 Human Kits. Data show the mean from *n* = 9 samples from three independent experiments. Differences were determined using ordinary one‐way ANOVA tests.

In the initial phase after viral infection, hypersecretion of cytokine can increase the risk of establishing viral infection.^[^ ^69^ ^]^ It is well known that genital inflammation can increase HIV‐1 acquisition in humans.^[^ ^35^, ^36^ ^]^ One proposed reason for the failure of microbicides to prevent HIV‐1 infection in clinical trials is the induction of inflammation in the genital mucosal epithelium. Resting T‐lymphocytes are naturally resistant to HIV‐1 infection but become permissive to HIV infection after activation by cytokines.^[^ ^70^ ^]^ We therefore studied the cytokine secretion from phytohemagglutinin (PHA) activated hPBMCs during HIV‐1 exposure with and without BSM gel protection. Secretion of pro‐ (TNF*α*, MCP‐1, IL‐1B, IL‐8) and anti‐inflammatory (IL1RA: IL‐1 inhibitor^[^ ^71^ ^]^) cytokines from hPBMCs was significantly dampened when cells were protected with BSM gels during HIV‐1 exposure (**Figure** **6**). Interestingly, treatment with BSM gels also significantly dampened the TNF*α* secretion from HIV‐1‐infected cells (Figure 6, HIV‐1 vs BSM gels HIV‐1). It is known that increased secretion of the proinflammatory cytokines IL‐6 and IL‐8 and the anti‐inflammatory IL‐1RA in cervicovaginal epithelial cells increases the risk of HIV‐1 acquisition.^[^ ^72^ ^]^ In addition, inhibition of MCP‐1 secretion from hPBMCs reduces HIV‐1 infection in individuals positive for HSV‐2 or cytomegalovirus CMV virus.^[^ ^73^ ^]^ More generally, cytokine production in genital tissue promotes recruitment and activation of various types of immune cells, such as T cells (CD4+, CCR5+, CXCR4+),^[^ ^74^ ^]^ macrophages,^[^ ^75^ ^]^ and dendritic cells (DCs).^[^ ^76^ ^]^ These immune cells are the primary targets of HIV‐1 and HSV‐2. So, we propose that suppression of cytokine secretion by BSM gels also contributes to prophylaxis of HIV‐1 infection.

## 3 Conclusion

In this study, we synthesized BSM gels that liquefied under large strain and self‐healed rapidly and repeatedly after the strain was removed. We showed that these properties were essential for mucin lubrication, as evidenced by significantly improved lubrication by the mucin gels (5%, wt/v) compared to mucin solutions (5%, wt/v). We also showed that the gels inhibited the infection of HIV‐1 and HSV‐2 in epithelial cells and immune cells by ≈70% and ≈80%, respectively. For HIV‐1, our data suggest that a combination of steric filtering, binding to the viral glycoprotein gp120, and attenuation of immune cell activation are responsible for the protective effect. HIV‐1/AIDS remains one the most serious global epidemic despite massive investments and investigation into its containment. Taken together, we developed a gel‐based lubricant that has significant potential use for the prophylaxis of individuals exposed to HIV‐1 and/or HSV‐2.

## 4 Experimental Section

### Materials

Mucin from bovine submaxillary glands (BSM, type I‐S), sodium periodate (ACS reagent, ≥99.8%), ethylene glycol (≥99%), sodium chloride (≥99%), 3‐Amino‐1,2‐propanediol (97%), acetonitrile (gradient grade, ≥99.9%), (ethylenediamine, 1‐cyano‐2‐ethoxy‐2‐oxoethylidenaminooxy) dimethylamino‐morpholino‐carbenium hexafluorophosphate (COMU, 97%), N,N‐diisopropylethylamine (DIPEA, ≥99%), and carbohydrazide (98%) were purchased from Sigma Aldrich. Other reagents, kits, and consumables are indicated below.

### Synthesis of BSM‐Aldehyde Derivatives

BSM was dissolved in MQ‐water at a concentration of 10 mg mL^−1^. 8 mmol COMU per gram of dry mucin was mixed with the BSM solution. DIPEA and 3‐amino‐1,2‐propanediol were dissolved at 16 mmol and 8 mmol per gram of dry mucin, respectively, in a 1:1 (v:v) solution of MQ‐water/acetonitrile. The mixture of DIPEA and 3‐amino‐1,2‐propanediol was added dropwise, stirred, and incubated overnight at 4 °C. Samples were then dialyzed in 100 kDa tubes (Float‐A‐Lyzer G2 from SpectrumLabs) at 4 °C against a 0.3 m NaCl solution for 2 d and MQ‐water for one extra day. Cis‐diol modified mucin samples were freeze‐dried and stored at −20 °C.

To synthesize the BSM aldehyde derivative, *cis*‐diol installed BSM was dissolved in MQ‐water at a concentration of 5 mg mL^−1^. Sodium periodate was added to give a final concentration of 1 × 10^−3^ m in the solution per 10 mg of dry *cis*‐diol‐installed BSM. Samples were protected from light using aluminium foil, and incubated at 4 °C for 15 min. The reaction was quenched by adding fivefold excess of ethylene glycol to sodium periodate in a molar ratio and incubated for 2 h at 4 °C. Samples were then dialyzed in 100 kDa tubes (Float‐A‐Lyzer G2 from SpectrumLabs) at 4 °C against a 0.3 m NaCl solution for 2 d and MQ‐water for one additional day. BSM‐aldehyde derivatives were freeze‐dried and stored at −20 °C.

### Synthesis of BSM‐Hydrazide Derivatives

BSM was dissolved in MQ‐water at a concentration of 10 mg mL^−1^. 8 mmol COMU per gram of dry mucin was mixed with the BSM solution. DIPEA and carbohydrazide were dissolved at 16 and 0.4 mmol per gram of dry mucin, respectively, in a 1:1 (v:v) solution of MQ‐water/acetonitrile. The mixture of DIPEA and carbohydrazide was added dropwise, stirred, and incubated overnight at 4 °C. Samples were then dialyzed in 100 kDa tubes (Float‐A‐Lyzer G2 from SpectrumLabs) at 4 °C against a 0.3 m NaCl solution for 2 d and MQ‐water for one additional day. BSM‐hydrazide derivatives were freeze‐dried and stored at −20 °C.

### Attenuated Total Reflectance‐Fourier Transform Infrared Spectroscopy

The ATR‐FTIR spectrum of unmodified BSMs, BSM‐*cis*‐diol, BSM‐aldehyde, BSM‐hydrazide derivatives, and BSM‐gels was studied (Perkin Elmer, UK). The ATR measuring mode, a diamond crystal with a contact area of 2 mm and a penetration depth of 2 µm, and a spectral resolution of 4 cm^−1^ at 16 spectra accumulations were used. All spectra were normalized, and baseline corrected (using OriginPro 8 software).

### Schiff Assay

The formation of BSM aldehydes was confirmed by Schiff assay, according to a published protocol^[^ ^41^ ^]^ in a 96‐well plate (Greiner, f‐bottom). 120 µL of a freshly prepared solution of 0.06% periodic acid in 7% acetic acid was added to a 25 µL mucin solution and mixed five times with a pipette. The plate was sealed with a plastic film and incubated for 1.5 h at 37 °C. The plate was then cooled to room temperature, and 100 µL Schiff's reagent was added and mixed with the pipette. The plate was resealed, shaken for 5 min, and the color was allowed to develop at room temperature. The plastic seal was removed, and absorbance was measured at 550 nm using a plate reader (Clario Star, BMG Labtech).

### Swelling/Disintegration Studies

BSM gels were prepared in 100 µL at a concentration of 50 mg mL^−1^, as described above. After 1 h, gels were removed from the mold and suspended in 500 µL of buffer with acidic (pH 5.5 acetate buffer), neutral (pH 7.4 PBS buffer), or basic (pH 9.0 PBS buffer) pH. For the enzymatic degradation experiment, BSM gels were suspended in 500 µL of pH 7.4 PBS buffer containing 2% neuraminidase (P0720S, 5 × 10^4^ U mL^−1^, New England Biolabs) or trypsin (50 µL of 0.5 µg mL^−1^, Sigma Alrich). After removing the supernatant, the weight of BSM gels was measured at different time points and replenished by fresh medium. The % weight (w/w) change was estimated using the following equation: % weight change = (*w*_1_−*w*_0_)/*w*_0_ × 100, where *w*_0_ is the weight of the gel before swelling and *w*_1_ is the weight of the gel after swelling.

### Rheological Characterization

The rheological properties (i.e., linear viscoelastic response, fluidization behavior at large strains, the self‐healing capacities) of the BSM gels were investigated using a commercial shear rheometer (MCR 302, Anton Paar, Graz, Austria) equipped with a plate‐plate geometry (bottom plate: P‐PTD 200/AIR, Anton Paar; 25 mm steel measuring head: PP25, 79044, Anton Paar). The sample volume was 100 µL for each measurement; the plate spacing was set to 0.15 mm and kept constant for all measurements. A solvent trap (a chamber with a water‐soaked paper towel, covered with a lid), as well as temperature control of the bottom plate (set to 21 °C), was used for all measurements. BSM aldehyde and hydrazide derivatives were dissolved in PBS buffer (Dulbecco's phosphate‐buffered saline, pH = 7.4, Sigma Aldrich) at 50 mg mL^−1^ each. Equal volumes of the BSM aldehyde and BSM hydrazide solutions were mixed and transferred to the rheometer bottom plate using a 100 µL positive‐displacement pipette (Rainin, Mettler Toledo, Columbus, USA). The measuring head was then lowered, and the test was started. Time‐dependent sweep analyses in torque‐controlled mode (0.5 µN m, constant oscillation frequency of 1 Hz) were performed to characterize the linear viscoelastic response of the gels, and the storage modulus (*G*′) and loss modulus (*G*″) were recorded every 7.5 s for 20 min. Afterward, a frequency sweep within the range of 0.1–10 Hz was determined in a strain‐controlled mode by applying a strain corresponding to a torque of ≈0.5 µN m. Subsequently, large amplitude oscillatory shear (LAOS) tests were applied to assess the onset of the nonlinear material response regime and to test for gel fluidization at large strain levels. Here, time‐dependent sweep analyses in strain‐controlled models were conducted using increasing oscillatory shear strain amplitudes (from 0.1% to 1000%) at a constant oscillation frequency of 1 Hz. Immediately after those strain‐ramps, time‐dependent measurements at low mechanical load (using a small constant torque of 0.5 µN m and a constant oscillation frequency of 1 Hz) were conducted to assess the self‐healing properties of the material, i.e., its ability to recover its initial viscoelastic properties after shear‐induced fluidization.

Both the average molecular weight^[^ ^77^ ^]^ (*M*_c_, the molecular weight of chain segments between two adjacent crosslinking or entanglement points) and the mesh size^[^ ^78^ ^]^ (◻, the distance between two adjacent crosslinking or entanglement points) were calculated according to the following equations

(1) $$Mc\;=\;\frac{c\rho RT}{G{{}^{\prime }}_{P}}$$

(2) $$\xi =\;{\left(\frac{G{{}^{\prime }}_{P}N_{A}}{RT}\right)}^{-1/3}$$

where *c* is the concentration of polymers (1.5 or 2.5% wt/v), *ρ* is the density of water at 298 K (997 kg m^−3^), *R* is the molar gas constant (8.3144598 × 10^6^ cm^3^ Pa K^−1^ mol^−1^), *T* is the temperature (298 K), $G{{}^{\prime }}_{P}$ is the plateau value of elastic modulus, and *N*_A_ is the Avogadro constant.

### Tribological Tests

BSM aldehyde and hydrazide derivatives (or unmodified BSM) were dissolved in PBS buffer (Dulbecco's phosphate‐buffered saline, pH = 7.4, Sigma Aldrich) at 50 mg mL^−1^, respectively. Equal volumes of the BSM aldehyde and hydrazide derivative solutions were mixed, and a total of 500 µL of the ensuing BSM gels (or a simple BMS solution) were transferred to a commercial shear rheometer (MCR 302, Anton Paar, Graz, Austria) equipped with a tribology unit (T‐PTD 200, Anton Paar) comprising a steel ball‐on‐PDMS cylinder geometry. The steel ball had a diameter of 12.7 mm (1.4301, *S*_q_ < 0.2 µm, Kugel Pompel, Vienna, Austria). PDMS cylinders with a diameter of 6.1 mm were prepared by mixing PDMS with a crosslinking reagent (Sylgard 184, Dow Corning, Wiesbaden, Germany) at a ratio of 10:1 (wt) and debubbling the mixture in a vacuum chamber. The solution was then poured into a cylinder‐shaped steel mold and cured at 80 °C for 4 h. The individual PDMS cylinders were inserted into the steel holder, and their height was adjusted with headless screws. The tribology measurements were performed at a constant normal force of *F*_N_ = 6 N.

This normal force was chosen such that friction in the boundary, mixed, and hydrodynamic regimes could be probed within the accessible speed range.^[^ ^79^ ^]^ Based on the Hertzian contact theory, the average contact pressure *p*_0_ was estimated using the following formula

(3) $$p_{0}=\frac{2}{3}p_{max}=\frac{2}{3\pi }\sqrt[{3}]{\frac{6F_{N,\;per\;pin}\;\times \;E^{{}^{\prime }2}}{R^{2}}}\text{with}\frac{1}{E^{\prime }}=\;\frac{1-v_{1}^{2}}{E_{1}}+\frac{1-v_{2}^{2}}{E_{2}}$$

Using the elastic modulus and Poisson's ratios of steel (*E*_steel_ = 210 GPa, *ν*_steel_ ≈ 0.30) and PDMS (*E*_PDMS_ ≈ 2 MPa, *ν*_PDMS_ ≈ 0.49),^[^ ^80^ ^]^ the Hertzian contact theory returned an average contact pressure of ≈0.31 MPa.

Before recording the first measuring point, the system was allowed to stabilize at a rotational speed of 700 mm s^−1^ for 30 s. Then, the speed‐dependent friction behavior was determined by performing a logarithmic speed ramp from 700 to 0.001 mm s^−1^. The friction coefficient was measured at 48 distinct speed levels for a duration of 10 s at each speed level. BSM solutions were compared. All tribological experiments were conducted at 21 °C and using a solvent trap. A paper towel soaked with water was used in a closed chamber to prevent evaporation of the sample during the experiments.

### Viral Infection Assay

HIV‐1 virus stock was obtained by transient transfection of pNL(AD8) plasmid (NIH AIDS Research and Reference Reagent Program, USA) into HEK‐293T cells (ATCC, USA). Supernatants were collected after 48 and 72 h. Viral stocks were purified by centrifugation before viral titer was determined using the HIV‐1 p24 ELISA kit (INNOTEST Innogenetics, Belgium).

HIV‐1 (R5‐HIV‐1_AD8_) infection in epithelial TZM.bl cells was studied. TZM.bl cells^[^ ^81^, ^82^ ^]^ (NIH AIDS Research and Reference Reagent Program, USA) were cultured in a DMEM medium containing 10% FBS in a humidified 37 °C incubator with 5% CO_2_. The medium was changed every second day. For the infection assay, TZM.bl cells were seeded at 10^4^ cells per well in a 96‐well plate and incubated for 24 h in a humidified 37 °C incubator with 5% CO_2_ to allow cell adhesion. BSM‐aldehyde and BSM‐hydrazide derivatives or unmodified BSM were dissolved in a PBS buffer to 50 mg mL^−1^. Then 25 µL of the solutions of BSM‐aldehyde derivatives and BSM‐hydrazide derivatives were added to the cells and mixed in a plate mixer to obtain BSM gel layers (50 µL). The same volume of BSM solutions and HEC gels was added to each well in the BSM solution group and the HEC gel group. 10 µL of RPMI medium without serum containing HIV‐1 was added (100 TCID50/well) and incubated for 2 h. BSM gels were removed by adding RPMI medium containing 10% FBS and incubating for 10 min and pipetting up and down generously. The removal of BSM gels was previously confirmed by Alcian blue staining. Then, 150 µL of DMEM medium containing 10% FBS was added to each well. After 48 h of incubation, HIV‐1 replication was quantified by measuring the luciferase activities (relative light units) using the Bright‐Glo Luciferase Assay System (Promega, USA).

HIV‐1 (R5‐HIV‐1_AD8_) infection in hPBMCs from healthy donors was studied. Cells were cultured in RPMI medium containing 10% FBS in a humidified 37 °C incubator with 5% CO_2_. The medium was changed every second day. Cells were incubated with the complete cell culture medium containing 2 µg mL^−1^ phytohemagglutinin (PHA, Sigma Aldrich) for 48 h to activate resting T‐lymphocytes.^[^ ^83^ ^]^ PHA‐treated cells were seeded in a 96‐well round‐bottom plate with 2 × 10^5^ cells per well. BSM‐aldehyde and BSM‐hydrazide derivatives or unmodified BSM were dissolved in a PBS buffer to 50 mg mL^−1^. Then 25 µL of the solutions of BSM‐aldehyde derivatives and BSM‐hydrazide derivatives were added to the cells and mixed in a plate mixer to obtain BSM gel layers (50 µL). The same volume of BSM solutions and HEC gels was added to each well in the BSM solution group and the HEC gel group. 10 µL of RPMI medium without serum containing HIV‐1 was added (100 TCID50/well) and incubated for 2 h. BSM gels were removed by adding RPMI medium containing 10% FBS and incubating for 10 min and pipetting up and down generously. The removal of BSM gels was previously confirmed by Alcian blue staining. The medium was replaced by centrifugation and aspiration. Cells were washed twice with PBS. Then, 150 µL of RPMI medium with 10% FBS was added to each well. Three days later, supernatants from HIV‐1‐infected hPBMCs were collected and 100 µL were added to TZM.bl cells preseeded the previous day in a 96‐well plate as described above. After 48 h of incubation, HIV‐1 replication was quantified by measuring luciferase activities (relative light units) using the Bright‐Glo Luciferase Assay System (Promega, USA). HIV‐1 capsid protein p24 was expressed in large in during the early stages of HIV‐1 infection.^[^ ^84^ ^]^ Thus, the p24 expression in HIV‐1‐infected hPBMCs was studied by quantifying p24 in cell lysates using an ELISA plate (p24 ELISA, Cat. No. 80564, INNOTEST HIV Antigen mAb), according to the manufacturer's instructions.

Herpes virus type II clinical isolate (HSV‐2) was expanded on VERO cells, titrated by plaque assay and stored at −80 °C. HSV‐2 infection in VERO cells, epithelial cells extracted from an African green monkey, was studied. Cells were seeded in a 24‐well plate with 175 × 10^3^ cells per well and incubated for 24 h to allow cell adhesion. The medium was aspirated, and 100 µL of BSM aldehyde and BSM hydrazide were added to the cells and mixed on a plate mixer to obtain BSM gel layers. BSM‐aldehyde and BSM‐hydrazide derivatives or unmodified BSM were dissolved in a PBS buffer to 50 mg mL^−1^. Then 25 µL of the solutions of BSM‐aldehyde derivatives and BSM‐hydrazide derivatives were added to the cells and mixed in a plate mixer to obtain BSM gel layers (50 µL). The same volume of BSM solutions and HEC gels was added to each well in the BSM solution group and the HEC gel group. 10 µL of RPMI medium without serum containing HIV‐1 was added (100 TCID50/well) and incubated for 2 h. BSM gels were removed by adding RPMI medium containing 10% FBS and incubating for 10 min and pipetting up and down generously. The removal of BSM gels was previously confirmed by Alcian blue staining. A DMEM medium without serum‐containing HSV‐2 was added (0.1 MOI) and incubated for 2 h. The medium with HSV‐2 was then discarded. Cells were washed twice with a PBS buffer. BSM gels were removed by the addition of DMEM with 10% FBS and incubating for 10 min. Cells were washed twice with PBS. Then, 1 mL DMEM medium containing 2% FBS and 0.7% low melting point agarose (LMP, Cat. No. 2070‐OP, Sigma Aldrich) was added to each well and incubated for 48 h in a humidified 37 °C incubator with 5% CO_2_. Viral plaques were counted and the percentage of plaques relative to plaques in control was calculated.

### Biolayer Interferometry Assay

The binding of BSM to gp120 was determined using biolayer interferometry on an Octet RED96 system. The system measures the increased mass of molecules on biosensors. The mass of gp120 (≈120 kDa) is much lower than that of BSMs (≈2000 kDa), thus, gp120 was immobilized on ARG2 biosensors (ForteBio, Octet RED 96, USA) to obtain stronger signals during real‐time binding measurements. Immobilization of gp120 was achieved by standard EDC‐NHS‐catalyzed amide bond formation to establish a covalent bond between reactive amines on gp120 and the carboxyl‐terminated biosensor surface. Black 96‐well polypropylene plates with flat bottoms were used for buffers and BSM solutions, with 200 µL per well.

The binding of BSM to gp120 was measured in a PBS buffer (pH 7.4) at 30 °C. The sensor tips were hydrated in the PBS buffer for 10 min before use. After hydration, gp120 was immobilized on the sensors in the next column of the plate containing the chemicals NHS/EDC and gp120. The unreacted carboxyl terminus on the biosensor surface was quenched by moving the sensors to the next column containing 1 m ethanolamine pH 8.5. After recording the baseline in PBS, all sensors were immersed in new columns with BSM solutions at different concentrations and the running buffer (used as the reference well) for 15 min to record the association kinetics. The sensors were then washed in wells containing a PBS buffer for 30 min to record dissociation kinetics. Data were analyzed by subtracting the shift reference sensors and wells using Octet Red analysis software. “2:1 heterogeneous ligand binding mode” was used to obtain the best fitting of the real‐time binding curve. The equilibrium dissociation constants (*K*_D, affinity_, *K*_a_ = 1/*K*_d_), association rate (*k*_a_) and dissociation rate (*k*_d_) constants, and the binding surface of BSM to HIV viral protein gp120 were calculated.

### Cytokine Quantification Assay

hPBMCs from healthy donors were incubated for 48 h in an RPMI medium supplemented with 10% FBS and 2 µg mL^−1^ PHA in a humidified 37 °C incubator with 5% CO_2_. PHA‐treated cells were seeded in a 96‐well round‐bottom plate with 2 × 10^5^ cells per well. The medium was replaced by centrifugation and aspiration. BSM‐aldehyde and BSM‐hydrazide derivatives or unmodified BSM were dissolved in a PBS buffer at the concentration of 50 mg mL^−1^, respectively. Then, 25 µL of BSM‐aldehyde and BSM‐hydrazide were added to the cells and mixed on a plate mixer to obtain BSM gel layers. HIV‐1 in an RPMI medium without serum was added (100 TCID50/well) and incubated for 2 h. The medium containing HIV‐1 was then discarded. Cells were washed twice with a PBS buffer. BSM gels were removed by adding RPMI with 10% FBS and incubating for 10 min. Cells were washed twice with PBS. Then, 150 µL of RPMI medium with 10% FBS was added to each well. One day later, the supernatants were collected, and cytokine production was analyzed using U‐PLEX Inflammatory Panel 1 Human Kit (Meso Scale Diagnostics, M.S.D., Cat. No. K15067L‐1), according to the manufacturer's instructions. Supernatants were centrifuged at 12 000 rpm for 10 min at 4 °C before being added to each precoated well. The fluorescent signals for detecting each cytokine were recorded using a Bio‐Plex 200 Array instrument (Bio‐Rad Laboratories), and data were analyzed using Bio‐Plex Manager software version 6.1.1., in comparison to the standard curve obtained for cytokine standards with known concentrations provided.

### Statistical Analysis

Statistical analysis was determined using GraphPad Prism 9.0, as indicated in the manuscript. *, **, ***, and **** indicate *P*‐values less than 0.05, 0.01, 0.0005, and 0.0001, respectively.

## Conflict of Interest

The authors declare no conflict of interest.

## Supporting information

Supporting Information

## Data Availability

The data that support the findings of this study are available from the corresponding author upon reasonable request.
