# Supplementary material for: Synthetic Mucin Gels with Self‐Healing Properties Augment Lubricity and Inhibit HIV‐1 and HSV‐2 Transmission
Source: Adv Sci (Weinh). 2022 Sep 14;9(32):2203898. doi: 10.1002/advs.202203898 (PMC9661867; doi:10.1002/advs.202203898)
Supplement: Supplementary file 1 — Supporting Information [file ADVS-9-2203898-s001.pdf]

ADVANCED SCIENCE
Open Access

Supporting Information

for *Adv. Sci.*, DOI 10.1002/advs.202203898

Synthetic Mucin Gels with Self-Healing Properties Augment Lubricity and Inhibit HIV-1 and HSV-2 Transmission

*Martin Kretschmer, Rafael Ceña-Diez, Cosmin Butnarasu, Valentin Silveira, Illia Dobryden, Sonja Visentin, Per Berglund, Anders Sönnerborg, Oliver Lieleg, Thomas Crouzier and Hongji Yan**

Supporting information

Synthetic Mucin Gels with Self-healing Properties Augment Lubricity and Inhibit HIV-1 and HSV-2 Transmission

Martin Kretschmer[2,3#], Rafael Ceña-Diez[4#], Cosmin Butnarasu[5], Valentin Silveira[6], Illia Dobryden[7], Sonia Visentin[5], Per Berglund[8], Anders Sönnerborg[4], Oliver Lieleg[2,3], Thomas Crouzier[1,8,9], Hongji Yan[1,8,9]*

[1]AIMES - Center for the Advancement of Integrated Medical and Engineering Sciences at Karolinska Institutet and KTH Royal Institute of Technology, Stockholm, Sweden

[2]Department of Mechanical Engineering and Munich School of Bioengineering, Technical University of Munich, Boltzmannstrasse 11, 85748 Garching, Germany

[3]Center for Protein Assemblies, Technical University of Munich, Ernst-Otto-Fischer Str. 8, 85748 Garching, Germany

[4]Department of Medicine Huddinge, Division of Infectious Diseases, Karolinska Institutet, I73, Karolinska University Hospital, 141 86 Stockholm, Sweden

[5]Department of Molecular Biotechnology and Health Science, University of Turin, 10135 Turin, Italy

[6]Department of Industrial Biotechnology, School of Engineering Sciences in Chemistry, Biotechnology and Health, KTH Royal Institute of Technology, AlbaNova University Center, 106 91 Stockholm, Sweden

[7]Division of Material and Surface Design, Department of Bioeconomy and Health, RISE Research Institutes of Sweden, Malvinas väg 3, SE-114 86, Stockholm, Sweden

[8]Department of Neuroscience, Karolinska Institutet, SE-171 77, Stockholm, Sweden

[9]Division of Glycoscience, Department of Chemistry, School of Engineering Sciences in Chemistry, Biotechnology and Health, KTH Royal Institute of Technology, AlbaNova University Center, 106 91 Stockholm, Sweden

# co-first author

*Corresponding author hongji.yan@ki.se and hongji@kth.se

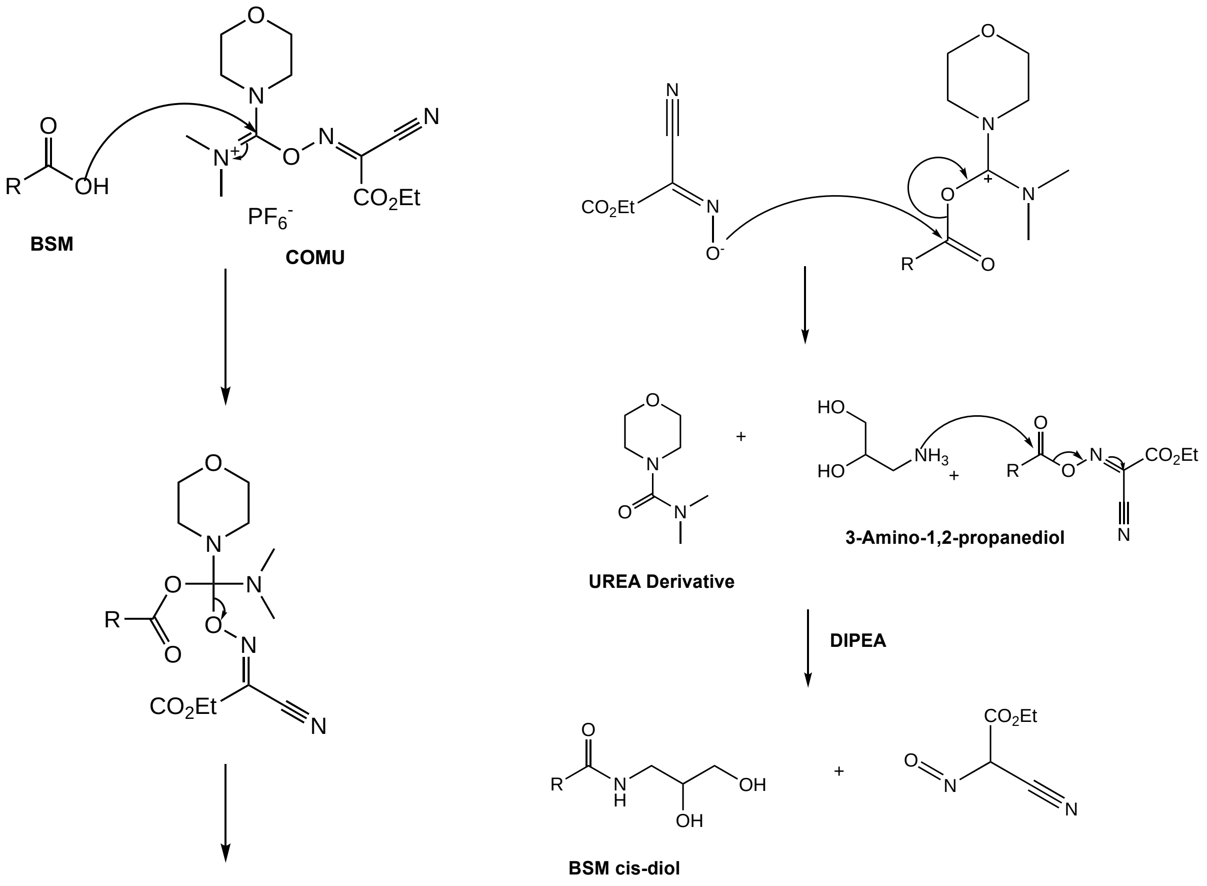

SI Figure 1. Synthesis of the BSM-cis-diol derivative.

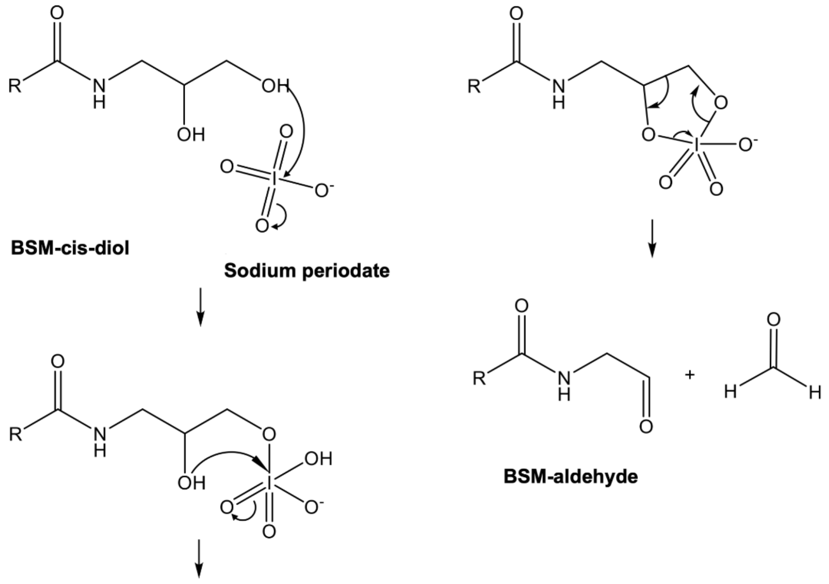

SI Figure 2. Synthesis of the BSM-aldehyde derivative.

Periodate oxidation kinetics of native BSM and aminoglycerol-modified BSM (BSM cis-diol) were studied by measuring the consumption of periodate over time[1]. The concentration of unreacted periodate

was determined from the standard curve ((0.1 to 1 mM/L)) with various known concentrations of periodate.

We used a one-phase decay model to fit the oxidation kinetic curve of BSM and BSM cis-diol derivative. In Figure S3 the red curve represents the fitting calculated according to the following equation:

$$y = p + Se^{-xk}$$

Where *p* represents the plateau, *S* is the span calculated as y_0 - *p*, and *k* is the rate constant expressed as the reciprocal of the *x* axis units. Half life was calculated as *ln*(2)/*k*.

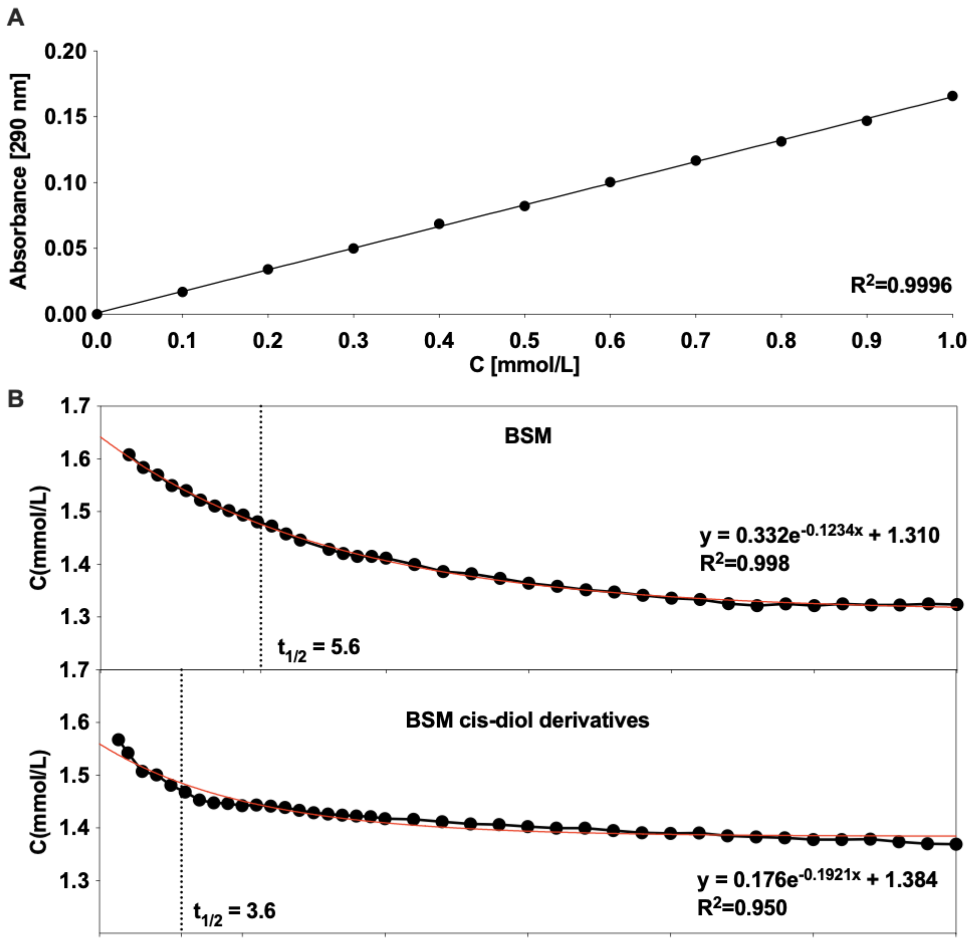

SI Figure 3. (A) Standard curve of periodate absorbance at 290 nm; (B) Kinetic plot for BSM and BSM-cis-diol derivatives.

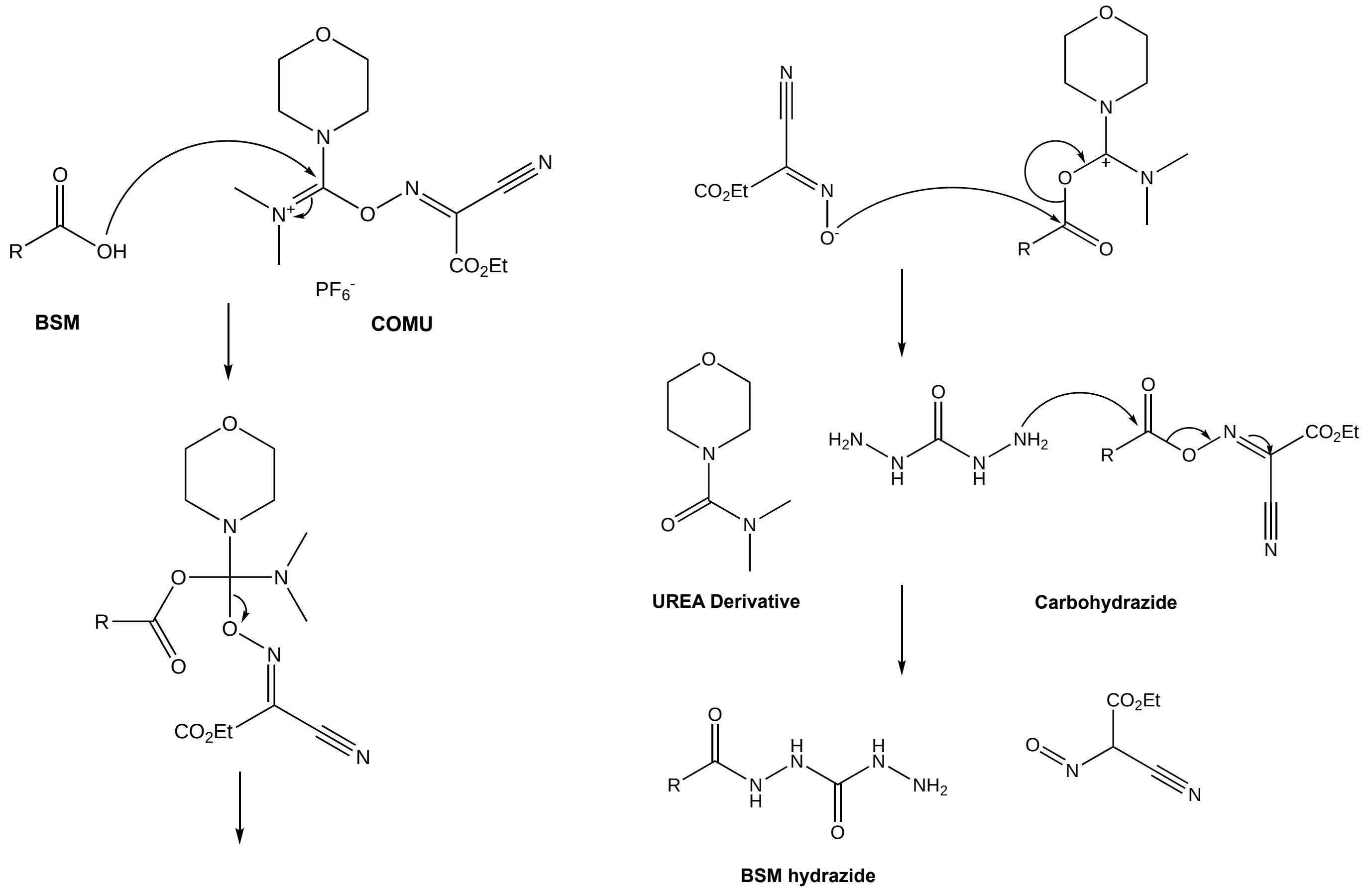

SI Figure 4. Synthesis of the BSM-hydrazide derivative.

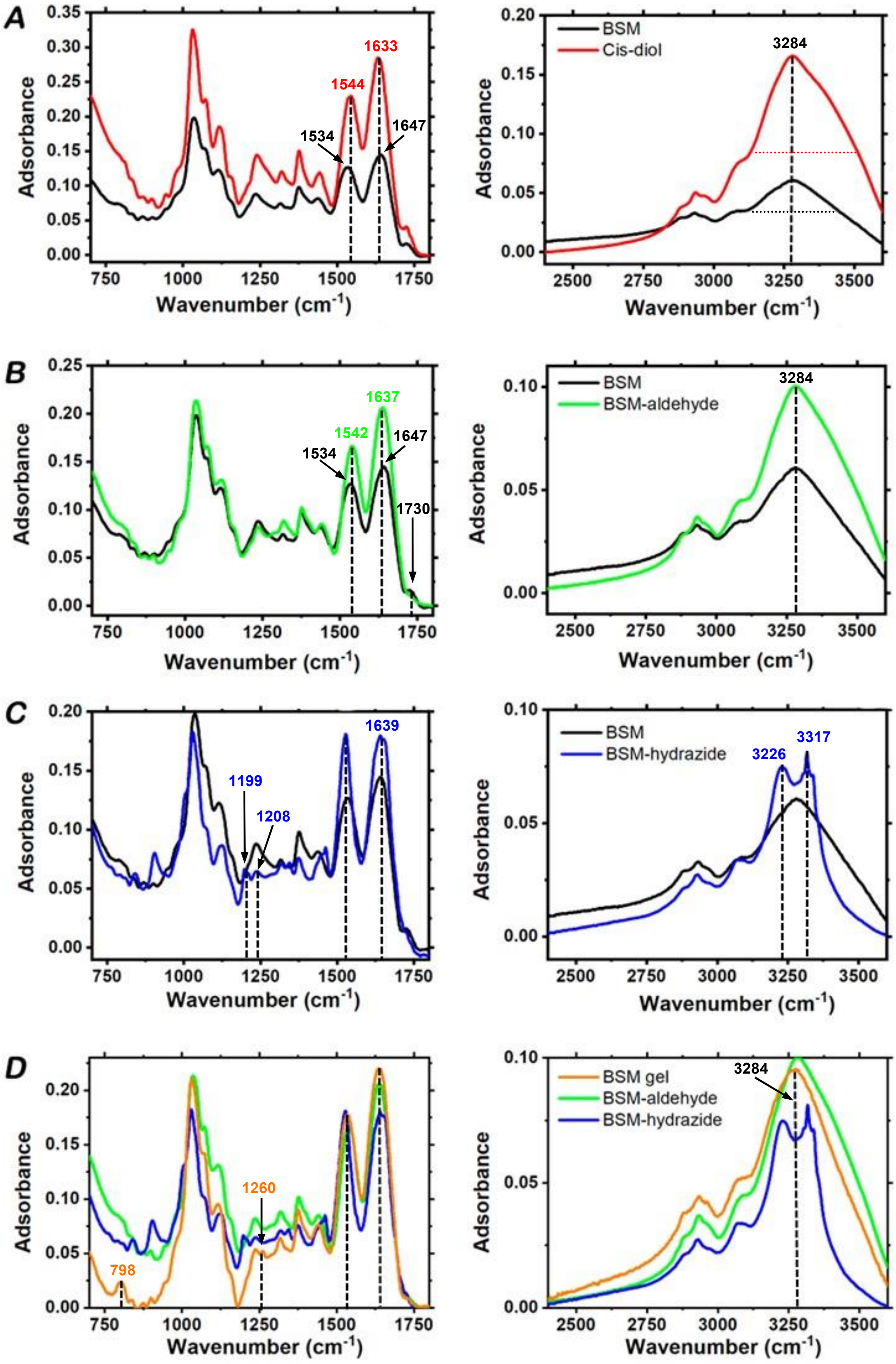
A
B
C
D
Adsorbance
Wavenumber (cm-1)
BSM
Cis-diol
BSM-aldehyde
BSM-hydrazide
BSM gel
1534
1544
1633
1647
3284
1542
1637
1730
1199
1208
1639
3226
3317
798
1260

SI Figure 5. The ATR-FTIR spectra of BSM-cis-diol (A, red), BSM-aldehyde (B, green), BSM-hydrazide derivatives (C, blue) compared to native BSMs (black lines). The ATR-FTIR spectrum of BSM gels (orange line) is compared to BSM-aldehyde (green line) and BSM-hydrazide derivatives (D, blue). While the entire measured spectra were from 700 cm^{-1} to 4000 cm^{-1}, the two magnified regions from 750 cm^{-1} to 1800 cm^{-1} (left column in panels A-D) and from 2400 cm^{-1} to 3600 cm^{-1} (right column in panels A-D) are shown in this figure. The dashed black lines are just a guide for specific characteristic peaks.

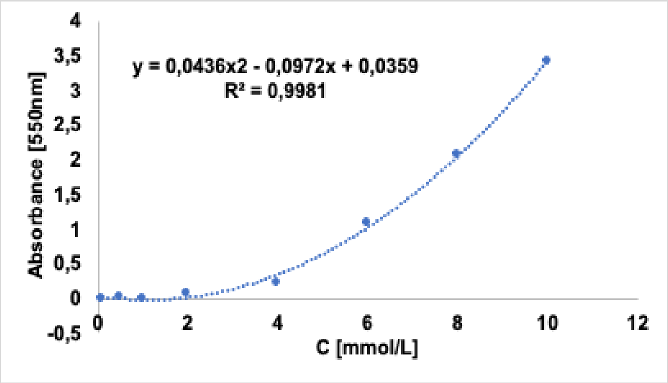

SI Figure 6. The standard curve for the quantification of aldehydes on BSM using a Schiff assay according to a published protocol[2]. The x-axis is the concentration of the aldehyde standards. The BSM aldehyde derivatives contain 9.34 ± 2.01 µmol aldehydes per mg.

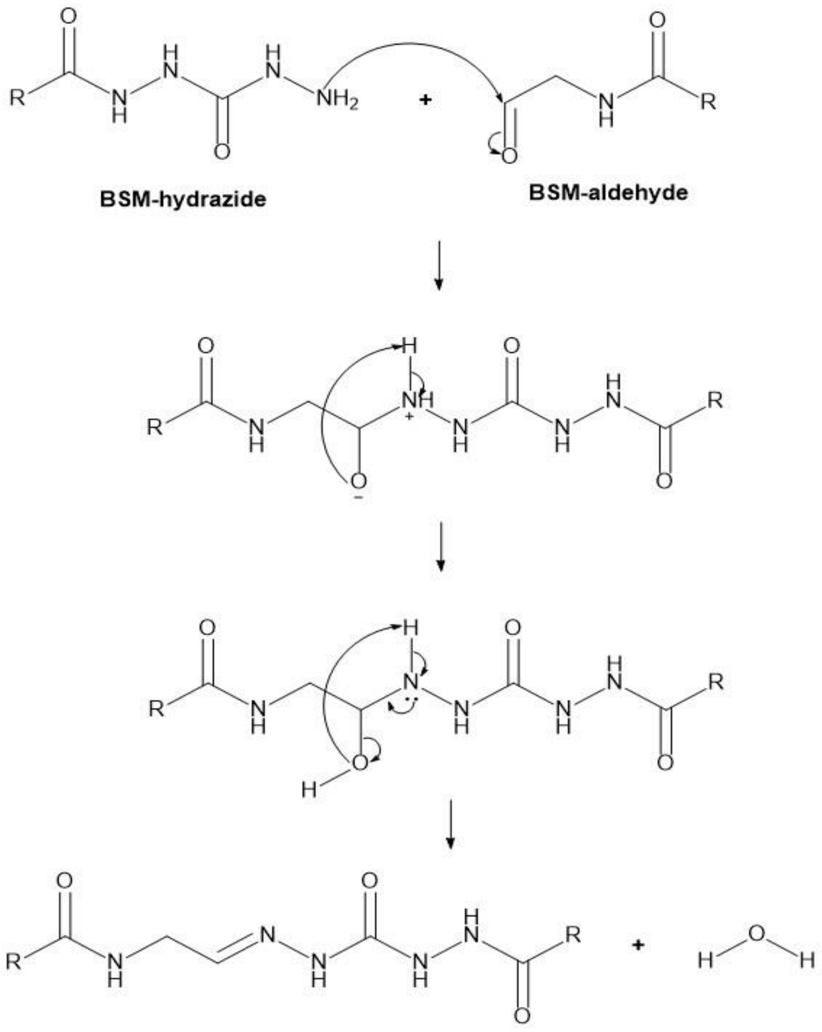

SI Figure 7. Hydrazone crosslinking reaction.

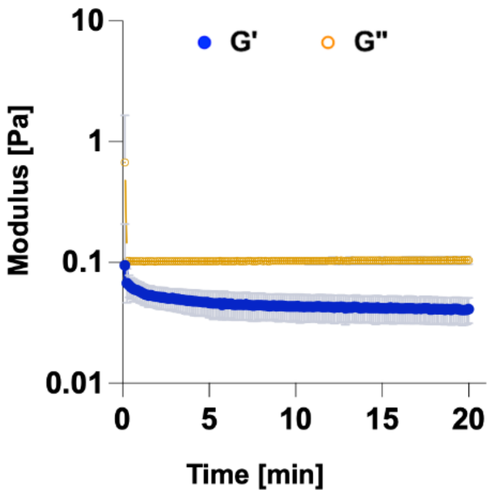

SI Figure 8. Time-dependent sweep analyses of BSM solution (wt/v). BSM solutions (5%, wt/v) are not gel-forming as indicated by the lower G’ than G’’ in the rheological measurements. The error bars denote the standard deviation obtained from n = 3 independent measurements.

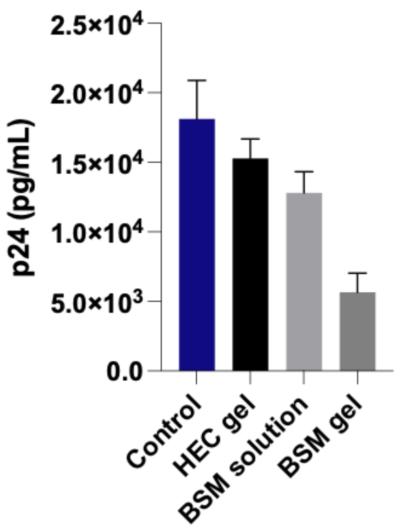

SI Figure 9. p24 production in HIV-1-infected hPBMCs with and without protection with HEC gels, BSM solution and BSM gels.

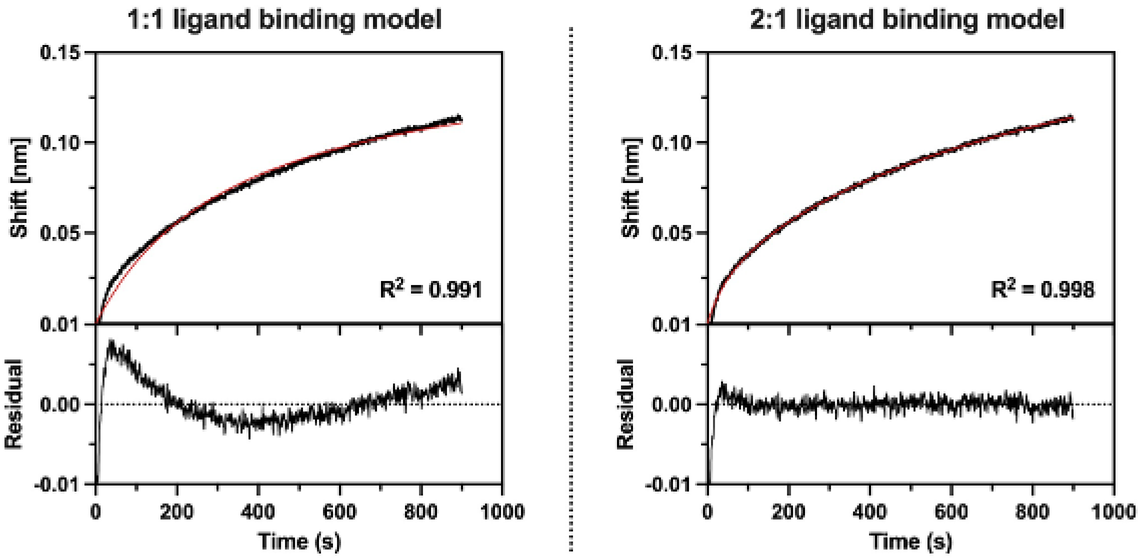

SI Figure 10. The fitting of the real-time binding curve of gp120 and mucin was analyzed using a '1:1 or 2:1 ligand binding model'

References:

[1] J. E. Scott, M. J. Tigwell, *Biochem. J* **1978**, *173*, 103.

[2] M. Kilcoyne, J. Q. Gerlach, M. P. Farrell, V. P. Bhavanandan, L. Joshi, *Analytical Biochemistry* **2011**, *416*, 18.
